# Health-related quality of life in patients with advanced melanoma treated with ipilimumab: prognostic implications and changes during treatment

**DOI:** 10.1016/j.esmoop.2022.100588

**Published:** 2022-09-16

**Authors:** E. Aamdal, E. Skovlund, K.D. Jacobsen, O. Straume, C. Kersten, O. Herlofsen, J. Karlsen, I. Hussain, A. Amundsen, A. Dalhaug, M. Nyakas, K.T. Hagene, K. Holmsen, S. Aamdal, S. Kaasa, T.K. Guren, J.A. Kyte

**Affiliations:** 1 Department of Oncology, Oslo University Hospital, Oslo, Norway; 2 Institute of Clinical Medicine, Faculty of Medicine, University of Oslo, Oslo, Norway; 3 Department of Public Health and Nursing, Norwegian University of Science and Technology, NTNU, Trondheim, Norway; 4 Department of Oncology and Medical Physics, Haukeland University Hospital, Bergen, Norway; 5 Centre for Cancer Biomarkers, Department of Clinical Science, University of Bergen, Bergen, Norway; 6 Research Unit, Sørlandet Hospital, Kristiansand, Norway; 7 Department of Oncology, Akershus University Hospital, Lørenskog, Norway; 8 Department of Oncology, Ålesund Hospital, Ålesund, Norway; 9 The Cancer Clinic, St. Olav’s Hospital, Trondheim University Hospital, Trondheim, Norway; 10 Department of Hematology and Oncology, Stavanger University Hospital, Stavanger, Norway; 11 Department of Oncology, University Hospital of North Norway, Tromsø, Norway; 12 Department of Oncology and Palliative Medicine, Nordland Hospital, Bodø, Norway; 13 Department of Cancer Immunology, Institute of Cancer Research, Oslo University Hospital, Oslo, Norway

**Keywords:** metastatic melanoma, health-related quality of life, ipilimumab, prognosis

## Abstract

**Background:**

We have previously reported that the safety and efficacy of ipilimumab in real-world patients with metastatic melanoma were comparable to clinical trials. Few studies have explored health-related quality of life (HRQL) in real-world populations receiving checkpoint inhibitors. This study reports HRQL in real-world patients receiving ipilimumab and assesses the prognostic value of patient-reported outcome measures.

**Patients and methods:**

Ipi4 (NCT02068196) was a prospective, multicentre, interventional phase IV trial. Real-world patients (*N* = 151) with metastatic melanoma were treated with ipilimumab 3 mg/kg intravenously as labelled. HRQL was assessed by the European Organisation of Research and Treatment of Cancer Quality of Life Questionnaire at baseline and after 10-12 weeks.

**Results:**

The European Organisation of Research and Treatment of Cancer Quality of Life Questionnaire was completed by 93% (141/151 patients) at baseline, and by 82% at 10-12 weeks. Poor performance status and elevated C-reactive protein (CRP) were associated with worse baseline HRQL. Clinically relevant and statistically significant deteriorations in HRQL from baseline to weeks 10-12 were reported (*P* <0.05). Baseline physical functioning [hazard ratio (HR) 1.96, *P* = 0.016], role functioning (HR 2.15, *P* <0.001), fatigue (HR 1.60, *P* = 0.030), and appetite loss (HR 1.76, *P* = 0.012) were associated with poorer overall survival independent of performance status, lactate dehydrogenase (LDH), and CRP. We further developed a prognostic model, combining HRQL outcomes with performance status, LDH, and CRP. This model identified three groups with large and statistically significant differences in survival.

**Conclusions:**

Systemic inflammation is associated with impaired HRQL. During treatment with ipilimumab, HRQL deteriorated significantly. Combining HRQL outcomes with objective risk factors provided additional prognostic information that may aid clinical decision making.

## Highlights

• HRQL was independently associated with survival in patients with metastatic melanoma receiving ipilimumab.
• Patients experienced clinically meaningful and statistically significant deteriorations in HRQL during therapy.
• A prognostic model combining HRQL and biological markers defined three groups with highly variable life expectancies.
• This prognostic model may be of use in clinical decision making.

## Introduction

The last decade has marked a therapeutic paradigm shift for patients with metastatic melanoma. Ipilimumab, an antibody targeting the cytotoxic T-lymphocyte-associated protein 4, was the first treatment to show improved overall survival (OS) in metastatic melanoma in a randomised phase III trial,^1^ followed by programmed cell death protein 1 (PD-1) inhibitors with or without ipilimumab, and BRAF inhibitors with or without MEK inhibitors.^2^ Despite therapeutic advances, life expectancy for patients with metastatic melanoma is still restricted.^2^ Thus, other treatment goals, such as preserving quality of life, are of great importance, and may affect benefit–risk assessment.

Health-related quality of life (HRQL) is defined by the Center for Disease Control and Prevention as ‘an individual’s or group’s perceived physical and mental health over time’,^3^ indicating a subjective, multidimensional evaluation of own well-being as opposed to a physician’s external, usually more disease-focused perspective. Factors shown to influence HRQL in cancer include weight loss, performance status, and systemic inflammation.^4^ Different tools for evaluating HRQL in patients with cancer exist, including more generic and more disease-specific questionnaires.^5^^,^^6^ Such patient-reported outcome measures (PROMs) have increasingly become an integrated part of clinical trials, acknowledging patients’ voice and recognising the valuable contributions of such measures.^7^

Few studies have explored HRQL in real-world populations receiving checkpoint inhibitors. Key HRQL issues in metastatic melanoma comprise pain; insomnia; fatigue; appetite loss; itching; nausea and vomiting; postsurgical symptoms; emotional distress; and restrictions in physical, role, and social functioning.^8^, ^9^, ^10^, ^11^ Reports from chemotherapy and interferon trials suggest worsening of HRQL during treatment.^10^

The Ipi4 trial was a prospective, phase IV trial providing ipilimumab to real-world patients with advanced melanoma. Treatment-associated high-grade toxicity was observed in 28% of patients, and immune-related adverse events (irAEs) in 56%.^12^ The median progression-free survival (PFS) was 2.7 months [95% confidence interval (CI) 2.6-2.8 months] and OS 12.1 months (95% CI 8.3-15.9 months), comparable to phase III trials. Poor performance status, elevated lactate dehydrogenase (LDH), and C-reactive protein (CRP) at baseline were independently associated with short survival. In this study, we aimed to investigate HRQL in patients with metastatic melanoma receiving ipilimumab. Further, we assessed how HRQL relates to laboratory and clinical markers, and if PROMs may be combined with such objective markers in a prognostic score.

## Patients and methods

### Patients and study design

The Ipi4 trial was a prospective, national, multicentre, single-armed, interventional phase IV trial (NCT02068196). Adult patients with histologically confirmed inoperable metastatic melanoma were included and received ipilimumab 3 mg/kg intravenously every 3 weeks for up to four doses. Key eligibility criteria included Eastern Cooperative Oncology Group performance status (ECOG PS) ≤1, no active brain metastases, autoimmune disease, or immunodeficiency. Any previous treatment was allowed. All patients provided written informed consent. The Ipi4 trial was approved by the Regional Committee for Medical and Health Research Ethics South East Norway and conducted in accordance with the ethical principles of the Declaration of Helsinki (1964).

### Study assessments

Toxicity was assessed by the Common Terminology Criteria for Adverse Events (CTCAE) version 4.0. Tumour response was evaluated by computed tomography using RECIST 1.1. Patients self-reported HRQL using the European Organisation of Research and Treatment of Cancer Quality of Life Questionnaire (EORTC-QLQ-C30) version 3.0.^5^ Questionnaires were completed before each visit at baseline; treatment weeks 4, 7, 10, and 12; and follow-up before progression. The EORTC-QLQ-C30 constitutes 30 questions that form subscales for global health (1), functioning (5), and symptoms (9, including 6 single measures and 3 scales) (13). A high score for global health or functioning scales represents high HRQL, while high scores for symptoms scales indicates more symptoms. Scores were converted according to the scoring manual.^13^ Based on the literature, deteriorations in global health, functional scales, fatigue, nausea and vomiting, pain, insomnia, and appetite loss were expected. Thus we decided to focus on these scales. Diarrhoea, being one of the most frequent and serious side-effects of ipilimumab, was also included in our analyses.

### Statistics

Statistical analyses were carried out using IBM SPSS Statistics for Windows, version 26 (IBM Corp., Armonk, NY, USA). Mean baseline HRQL was reported with 95% CIs and standard deviations. Mean scores in population subgroups were compared with one-way analysis of variance. The association between HRQL outcomes and baseline characteristics was tested using Pearson’s chi-square test, or the chi-square test for linear trend as appropriate.

Changes in HRQL during treatment were estimated by the mean difference between outcomes at baseline and 10-12 weeks. The week 12 assessment was preferred if both weeks 10 and 12 were available. A difference of ≥10 points in the scale of 0-100 was considered clinically meaningful,^14^ and *P* <0.05 was considered statistically significant. In addition, a mixed linear model including all available patient-reported outcomes from all patients (*N* = 144) at baseline to week 12 was used to investigate the robustness of the findings in the complete case analysis, constituted by the 102 patients who completed questionnaires at both baseline and weeks 10-12.

Survival was estimated by Kaplan–Meier analyses. OS was defined as time from treatment initiation to death, and PFS as time from treatment initiation to objective tumour progression or death. Patients were followed for at least 5 years or until death. Patients without an event were treated as censored 1 June 2020.

The effect of HRQL variables on OS was analysed using univariable and multivariable Cox proportional hazard modelling, reported as hazard ratios (HRs) with 95% CI. A cut-off at ≤66.7 was used for global health (average score 5/7). The average score of ‘a little’ (2/4) was used as cut-off for functioning scales (≤66.7) and for symptoms scales (≥33.3). A stepwise approach was applied to identify HRQL variables that were independently associated with OS in a Cox model including baseline ECOG PS, LDH, and CRP as covariates, factors previously identified as independent predictors of OS in the Ipi4 trial.^12^ HRQL variables with *P* <0.05 in unadjusted analyses were tested one-by-one in adjusted Cox models. Variables included in the final Cox model were checked for multicollinearity by the variance inflation factor and accepted if <3.

## Results

### Patient characteristics

From January 2014 to March 2015, 151 patients were included. At baseline, 93% (141/151) completed the questionnaire (Supplementary Figure S1, available at https://doi.org/10.1016/j.esmoop.2022.100588). Between weeks 10 and 12, 102 patients completed the questionnaire, constituting 72% (102/141) of patients who completed the baseline questionnaire and 82% (102/125) of patients alive at week 10. The proportion of patients with ECOG ≥1, LDH >upper limit of normal (ULN), and CRP ≥10 mg/l was marginally lower in the group completing both questionnaires; otherwise baseline characteristics were equally distributed (Table 1).

**Table 1:** Patient baseline characteristics

Characteristics	All patients^a^ (*N* = 141)	Patients responding at week 10-12^b^ (*N* = 102)
Age, years		
Median (range)	63 (27-84)	62 (29-81)
Sex, *n* (%)		
Female	53 (38)	39 (38)
Male	88 (62)	63 (62)
ECOG PS, *n* (%)		
0	104 (74)	80 (78)
1	35 (25)	22 (22)
≥2	2 (1)	0 (0)
M-stage, *n* (%)		
M1a^c^	15 (11)	14 (14)
M1b	22 (16)	18 (18)
M1c	92 (65)	63 (62)
M1d	12 (9)	7 (7)
*BRAF* status^d^, *n* (%)		
Mutated	65^e^ (46)	48^f^ (47)
Wild type	72^e^ (51)	52^f^ (51)
LDH^g^, *n* (%)		
≤ULN	76 (54)	62 (63)
>ULN	62 (44)	37 (36)
CRP^g^, *n* (%)		
<10 mg/l	86 (61)	68 (67)
≥10 mg/l	51 (36)	30 (29)
Prior therapy^h^, *n* (%)		
0	93 (66)	65 (64)
1	33 (23)	26 (26)
≥2	15 (11)	11 (11)

CRP, C-reactive protein; ECOG PS, Eastern Cooperation Oncology Group performance status; LDH, lactate dehydrogenase; M-stage, metastatic stage according to TNM versus 8; ULN, upper limit of normal at cut-off 205 U/l.

a All patients who replied to the baseline questionnaire.

b Patients who replied to questionnaires at baseline and week 10 or 12.

c Including one patient with M0.

d *BRAF*^*V600*^ genotype.

e Four patients not available.

f Two patients not available.

g Three patients not available.

h Systemic treatments.

### Baseline health-related quality of life

Baseline HRQL is outlined in Table 2. Patients completing both questionnaires generally reported more favourable HRQL outcomes compared with those replying only to the first questionnaire. The difference in scores was statistically significant and clinically meaningful for global health (78.3 versus 65.8), role functioning (85.0 versus 66.2), social functioning (83.3 versus 71.8), and fatigue (22.0 versus 35.6), compared with patients completing only the baseline questionnaire. Baseline HRQL by patient subgroups is presented in Supplementary Tables S1 and S2, available at https://doi.org/10.1016/j.esmoop.2022.100588. In patients aged ≥65 versus <65 years, no significant difference was observed apart from role functioning (86.0 versus 75.6). Patients with ECOG PS ≥1 reported clinically and statistically significantly worse baseline scores for global health (58.6 versus 80.5), all functioning scales, fatigue (42.3 versus 19.9), pain (39.2 versus 10.3), and appetite loss (24.3 versus 10.3). Brain involvement was associated with a clinically meaningful and significantly higher score for appetite loss (*P* = 0.043); otherwise metastatic stage did not confer significant differences in HRQL. Patients with *BRAF*^*V600*^ mutation reported statistically significantly more pain (23.1 versus 13.2), and poorer role functioning than patients who were wild type (75.6 versus 84.5). Baseline HRQL seemed independent of LDH level. When compared with patients with CRP <10 mg/l, patients with CRP ≥10 mg/l scored statistically and clinically significantly worse for global health (68.0 versus 78.5), role functioning (70.3 versus 84.9), social functioning (69.9 versus 85.7), fatigue (34.9 versus 20.9), pain (29.4 versus 11.4), and appetite loss (25.5 versus 7.8). A significantly higher proportion of patients with CRP ≥10 mg/l had poor ECOG PS (37% versus 17%) and elevated LDH (59% versus 35%) at baseline compared with those with CRP <10 mg/l.

**Table 2:** Baseline health-related quality of life^a^

Health-related quality of life	All patients completing baseline questionnaire (*N* = 141)			Patients completing baseline questionnaire only (*N* = 39)			Patients completing both questionnaires (*N* = 102)			*P*-value^c^
	Mean	95% CI	SD	Mean	95% CI	SD	Mean	95% CI	SD	
Global health/QoL	74.9^b^	71.3-78.5	21.6	65.8^b^	58.3-73.3	22.7	78.3	74.3-82.2	20.2	0.002
Physical functioning	87.5	84.7-90.2	16.6	80.5	73.6-87.5	21.4	90.1	87.5-92.8	13.6	0.002
Role functioning	79.8	75.5-84.1	26.0	66.2	56.3-76.2	30.7	85.0	80.6-89.3	22.1	<0.001
Emotional functioning	83.1	80.0-86.2	18.9	80.6	74.5-86.6	18.6	84.1	80.3-87.8	19.0	0.324
Cognitive functioning	91.6	89.2-94.0	14.6	88.5	82.4-94.6	18.8	92.8	90.4-95.3	12.5	0.114
Social functioning	80.1	76.2-84.0	23.4	71.8	62.2-81.4	29.7	83.3	79.5-87.2	19.8	0.008
Fatigue	25.8	21.9-29.7	23.4	35.6	26.2-45.0	29.1	22.0	18.1-25.9	19.7	0.002
Nausea and vomiting	4.6	3.1-6.1	9.2	9.0	5.1-12.9	12.0	2.9	1.5-4.4	7.2	<0.001
Pain	17.9	13.6-22.1	25.6	22.6	13.5-31.8	28.2	16.0	11.2-20.8	24.5	0.170
Insomnia	19.9	15.5-24.3	26.4	26.5	16.5-36.5	30.8	17.3	12.6-22.1	24.3	0.065
Appetite loss	13.9	9.7-18.2	25.2	20.5	11.0-30.0	29.2	11.4	6.9-16.0	23.2	0.056
Diarrhoea	11.1	7.8-14.4	19.8	15.4	7.6-23.2	24.0	9.5	6.0-13.0	17.8	0.113

CI, confidence interval; QoL, quality of life; SD, standard deviation.

a EORTC QLQ-C30.

b One patient not available.

c ANOVA comparing patients completing both questionnaires with patients completing only baseline.

### Changes in health-related quality of life

**Figure 1:**
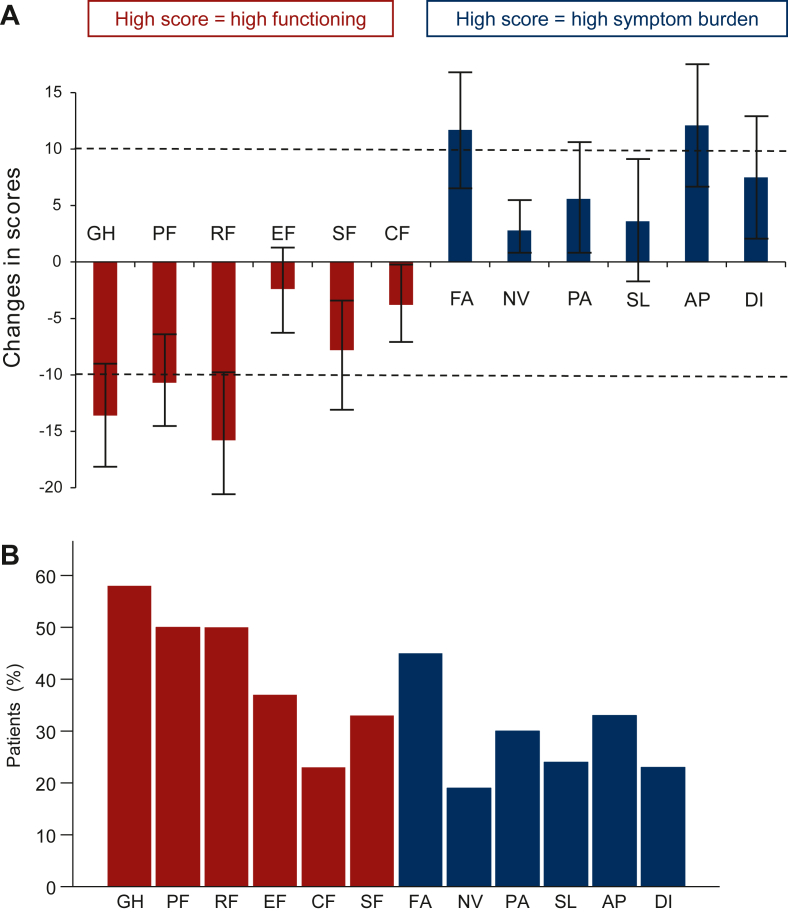
**Changes in health-related quality of life (HRQL) from baseline to weeks 10-12.** (A) Mean changes in HRQL outcomes from baseline to weeks 10-12 (*N* = 102). Functioning scales are shown in red and symptoms scales in blue. Dashed lines indicate cut-offs for clinical significance defined as a mean change from baseline ≥10. The mean change in global health (GH) was –13.6 (95% CI –18.1 to –9.1), physical functioning (PF) –10.7 (95% CI –14.7 to –6.7), role functioning (RF) –15.4 (95% CI –20.8 to –9.9), emotional functioning (EF) –2.5 (95% CI –5.9 to 1.0), social functioning (SF) –7.8 (95% CI –12.7 to –3.0), cognitive functioning (CF) –3.8 (95% CI –7.3 to –0.2), fatigue (FA) 11.4 (95% CI 6.4-16.4), nausea and vomiting (NV) 2.8 (95% CI 0.5-5.1), pain (PA) 5.6 (95% CI 0.6-10.5), insomnia (SL) 3.6 (95% CI –2.0 to 9.2), appetite loss (AP) 12.1 (95% CI 6.3-17.9), and diarrhoea (DI) 7.5 (95% CI 1.4-13.6). (B) Proportions of patients experiencing clinically meaningful deteriorations in HRQL from baseline to weeks 10-12; 58% of patients experienced a worsening in GH, 51% in PF, 50% in RF, 37% in EF, 23% in CF, 33% in SF, 45% in FA, 19% in NV, 30% in PA, 24% in SL, 33% in AP, and 23% in DI. CI, confidence interval.

The mean changes in HRQL from baseline to weeks 10-12 are presented in Figure 1A. Clinically meaningful and statistically significant deteriorations were observed for global health (–13.6, 95% CI –18.1 to –9.1), physical functioning (–10.7, 95% CI –14.7 to –6.7), role functioning (–15.4, 95% CI –20.8 to –9.9), fatigue (11.4, 95% CI 6.4-16.4), and appetite loss (12.1, 95% CI 6.3-17.9). No mean improvements were identified. Proportions of patients experiencing impairments in HRQL are shown in Figure 1B. Estimated changes from a model comprising data from all questionnaires (*N* = 144) are shown in Supplementary Table S3, available at https://doi.org/10.1016/j.esmoop.2022.100588. The results support findings from the complete case analysis.

Supplementary Tables S4 and S5, available at https://doi.org/10.1016/j.esmoop.2022.100588, depict changes in HRQL by subgroups. The mean reduction in global health was clinically and statistically significantly larger in patients considered ECOG PS 0 versus ECOG PS ≥1 (–16.3 versus –3.8). In comparison to patients with baseline LDH ≤ULN, those with baseline LDH >ULN experienced clinically and statistically significantly larger mean deteriorations in global health (–20.5 versus –9.8) and fatigue (19.2 versus 7.1). Treatment-naïve patients more commonly suffered deteriorations in global health (68% versus 41%) and fatigue (54% versus 30%) than pretreated patients. Changes in HRQL seemed independent of sex, metastatic stage, *BRAF*^*V600*^ mutation status, and CRP. No statistically significant association between subgroups and increase in diarrhoea was observed.

A numerical difference was noted for change in mean global health between patients who did and did not experience treatment-associated high-grade toxicity (–21.5 versus –12.5; *P* = 0.201), and patients who did or did not experience irAEs (–15.4 versus –11.5; *P* = 0.385) within the second questionnaire. However, this was not formally statistically significant.

### Baseline health-related quality of life and survival

**Figure 2:**
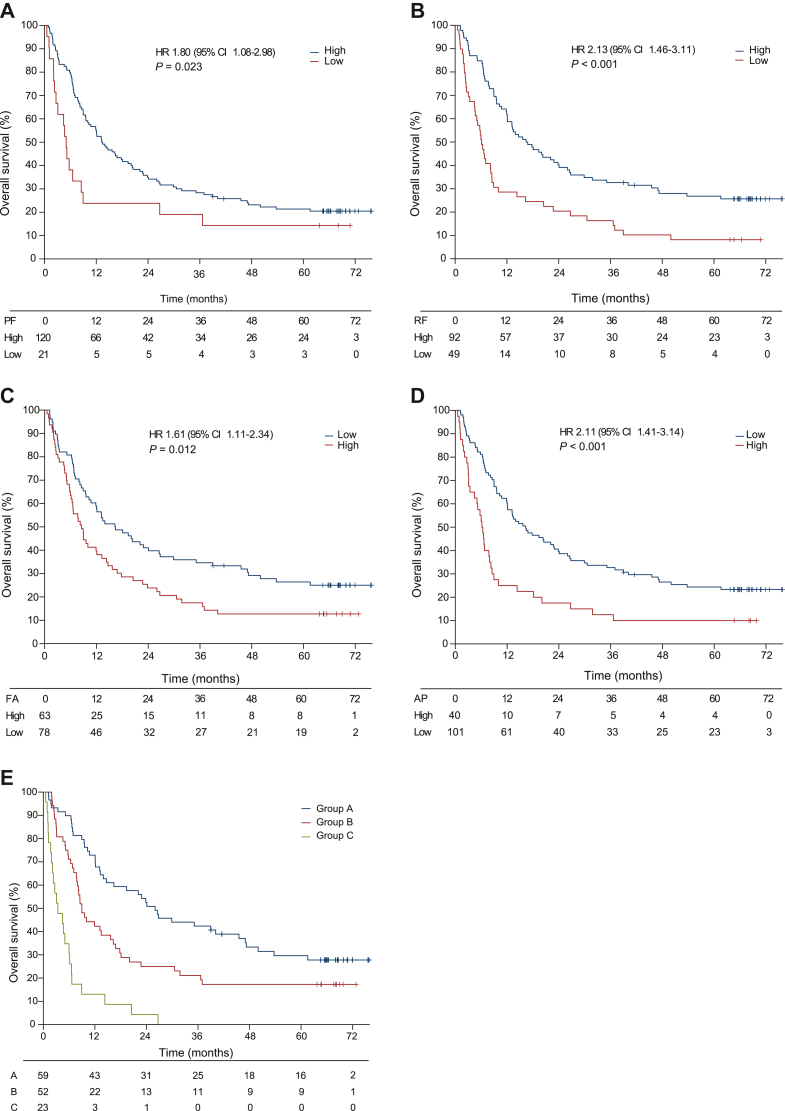
**Kaplan–Meier estimates for overall survival (OS) according to baseline health-related quality of life (HRQL).** Kaplan–Meier estimates for OS according to HRQL. (A) Physical functioning (PF). In patients who reported ‘a little’ or more impairment in PF (≤66.7), median OS was 5.0 months (95% CI 3.9-6.2 months) versus 13.3 months (95% CI 9.4-17.2 months) in patients who reported less or no impairment (>66.7). (B) Role functioning (RF). In patients who reported ‘a little’ or more impairment in RF (≤66.7), median OS was 6.1 months (95% CI 4.9-7.4 months), compared with 16.4 months (95% CI 10.0-22.8 months) in patients that reported less or no impairment (>66.7). (C) Fatigue (FA). In patients who reported ‘a little’ or more FA (≥33.3), median OS was 8.5 months (95% CI 6.0-11.1 months), and in patients who reported less or no FA (<33.3) median OS was 16.3 months (95% CI 8.8-23.8 months). (D) Patients who reported ‘a little’ or more appetite loss (AP; ≥ 33.3) had a median OS of 6.1 months (95% CI 4.9-7.4 months) versus 16.3 months (95% CI 9.9-22.7 months) in patients who reported less or no AP (<33.3). (E) Prognostic model combining patient-reported outcome measures with biomarkers. OS according to number of identified risk factors; Eastern Cooperative Oncology Group performance status (ECOG PS) ≥1, lactate dehydrogenase (LDH) >ULN, C-reactive protein (CRP) ≥10 mg/l, and impaired PF, RF, FA, and AP. Patients in group A (*N* = 59) harboured no risk or one factor, group B (*N* = 52) two to four, and group C (*N* = 23) five or more. Patients in group A lived for a median of 26.0 months (95% CI 17.6-34.4 months), patients in the group B lived for a median of 9.0 months (95% CI 6.9-11.1 months), and patients in group C lived for a median of 3.4 months (95% CI 0.6-6.2 months). CI, confidence interval; HR, hazard ratio; ULN, upper limit of normal.

Table 3 shows the association between baseline HRQL and OS. Global health (HR 1.57, *P* = 0.020), physical functioning (HR 1.80, *P* = 0.023), role functioning (HR 2.13, *P* < 0.001), social functioning (HR 1.51, *P* = 0.035), fatigue (HR 1.61, *P* = 0.012), pain (HR 1.50, *P* = 0.042), and appetite loss (HR 2.11, *P* < 0.001) were significantly associated with OS (Figure 2). In a Cox multiple regression model, adjusting for ECOG PS, LDH, and CRP, physical functioning (HR 1.96, *P* = 0.016), role functioning (HR 2.15, *P* < 0.001), fatigue (HR 1.60, *P* = 0.030), and appetite loss (HR 1.76, *P* = 0.012) were independently associated with a shorter OS. The association between baseline HRQL and PFS largely supported our findings for OS (Supplementary Table S6, available at https://doi.org/10.1016/j.esmoop.2022.100588).

**Table 3:** Cox proportional hazard modelling

Baseline characteristics and biomarkers		Univariable (*N* = 141)			Multivariable (*N* = 134)											
		HR	95% CI	*P*-value	HR	95% CI	*P*-value	HR	95% CI	*P*-value	HR	95% CI	*P*-value	HR	95% CI	*P*-value
ECOG PS ≥1 versus 0^a^		1.59	1.05-2.40	0.029	1.59	1.01-2.49	0.046	1.37	0.87-2.18	0.179	1.48	0.93-2.37	0.100	1.82	1.18-2.80	0.007
LDH >ULN versus ≤ULN^b^		2.38	1.63-3.48	<0.001	2.34	1.57-3.47	<0.001	2.30	1.55-3.42	<0.001	2.43	1.64-3.59	<0.001	2.31	1.55-3.44	<0.001
CRP ≥10 mg/l versus <10 mg/l^c^		1.98	1.35-2.90	0.001	1.66	1.12-2.47	0.012	1.58	1.05-2.36	0.028	1.59	1.07-2.36	0.022	1.38	0.90-2.12	0.143
Functioning scales^d^	≤66.7/>66.7^e^															
Global health/QoL^a^	38/61	1.57	1.07-2.29	0.020												
Physical functioning	15/85	1.80	1.08-2.98	0.023	1.96	1.14-3.38	0.016									
Role functioning	35/65	2.13	1.46-3.11	<0.001				2.15	1.40-3.28	<0.001						
Emotional functioning	20/80	1.21	0.76-1.93	0.429												
Social functioning	36/64	1.51	1.03-2.21	0.035												
Cognitive functioning	11/89	1.09	0.60-1.98	0.783												
Symptoms scales^f^	≥33.3/<33.3^g^															
Fatigue	45/55	1.61	1.11-2.34	0.012							1.60	1.05-2.44	0.030			
Nausea and vomiting	4/96	1.72	0.63-4.67	0.288												
Pain	31/69	1.50	1.02-2.23	0.042												
Insomnia	44/56	1.39	0.96-2.01	0.084												
Appetite loss	28/72	2.11	1.41-3.14	<0.001										1.76	1.13-2.74	0.012
Diarrhoea	27/73	1.14	0.75-1.73	0.541												

CI, confidence interval; CRP, C-reactive protein; ECOG PS, Eastern Cooperation Oncology Group performance status; HR, hazard ratio; HRQL, health-related quality of life; LDH, lactate dehydrogenase; ULN, upper limit of normal (205 U/L).

a One patient not available.

b Three patients not available.

c Four patients not available.

d Score ≤66.7 versus >66.7.

e Percentages of patients with HRQL ≤66.7/>66.7.

f Score ≥33.3 versus <33.3.

g Percentages of patients with HRQL ≥33.3/<33.3. Patients with missing data were excluded from analysis.

### Prognostic model combining biological factors and patient-reported outcome measures

As PROMs offer prognostic information, we developed a model combining HRQL outcomes with objective markers independently predicting a worse OS in this population (Figure 2E). This model comprised ECOG PS, LDH, CRP, physical functioning, role functioning, fatigue, and appetite loss. Three distinctive prognostic groups were identified. The risk of death was significantly increased in patients with five to seven risk factors (group C; HR 5.81, *P* < 0.001) and two to four risk factors (group B; HR 1.72, *P* = 0.013), compared with patients with zero or one risk factor (group A). Large differences in median survival was observed between the groups, with an eightfold advantage for group A compared with group C (26.0 months versus 3.4 months). No patients in group C survived >27 months. A prognostic model for PFS identified a group of patients with significantly poorer PFS, and thus, supported our findings for OS (Supplementary Figure S2, available at https://doi.org/10.1016/j.esmoop.2022.100588).

## Discussion

This study reported on HRQL in real-world patients with metastatic melanoma receiving ipilimumab. Baseline HRQL was comparable to the general population.^15^ The relatively high baseline HRQL in this study may partly be related to the hope of starting a new, promising treatment.^16^ Moreover, the psychological phenomenon known as response shift, causing patients’ conceptions and expectations of own HRQL to readjust during the disease course,^17^ may have contributed.

Baseline HRQL was generally poorer in patients only replying to the first questionnaire. These patients were more frequently reported with ECOG PS ≥1, LDH >ULN, and CRP ≥10 mg/l, factors associated with poorer OS in this trial.^12^ Previously, poor health has been recognised as a likely cause of patients not replying to questionnaires.^18^ This is a common issue in HRQL research and challenges generalisability of the results.

ECOG PS ≥1 was associated with clinically and statistically significantly worse mean scores for global health, functioning scores, fatigue, pain, and appetite loss. As ECOG PS is the physician’s grading of a patient’s ability to carry out daily activities, this finding is expected. ECOG PS is widely recognised to determine HRQL.^4^

Patients with *BRAF*^*V600*^ mutations reported significantly more pain and poorer role functioning at baseline, but no significant difference in global health. Impaired HRQL may be related to rapid tumour growth driven by the *BRAF* mutation, and consequently, a poorer prognosis, in line with results from a meta-analysis.^19^ A phase III trial, comparing treatment with BRAF inhibitors versus combined treatment with BRAF and MEK inhibitors in patients with *BRAF-*mutated metastatic melanoma, did indeed find that pain was a major HRQL issue in this population.^20^^,^^21^ Besides, in patients with colorectal cancer, *BRAF*-mutated disease has been associated with generally decreased HRQL.^22^ Increased inflammation, indicated by CRP ≥10 mg/l, was associated with clinically and statistically significantly poorer HRQL including global health, role functioning, social functioning, fatigue, pain, and appetite loss. The influence of systemic inflammation on HRQL and cancer-related symptoms including pain, anorexia, and fatigue has been previously recognised in advanced cancer.^4^^,^^23^^,^^24^ Moreover, CRP ≥10 mg/l was associated with a worse OS in this trial.^12^ Thus, in this patient population, even a mildly elevated CRP negatively affected the present by impairing HRQL, and the future by shortening OS.

We have previously reported that efficacy and toxicity of ipilimumab in the real-world setting were comparable to phase III trials.^12^ This also applies to the observed changes in HRQL. Clinically meaningful worsening in HRQL in patients receiving ipilimumab monotherapy was detected in the MDX010-20^25^ and KEYNOTE-006^26^ trials, with similar findings reported in an observational study in real-world patients with metastatic melanoma.^27^ The CheckMate 067 trial,^28^ however, noted no clinically meaningful changes in the ipilimumab arm, but compliance was <70% at treatment completion.

Contradicting previous findings,^25^ we did not detect that age >65 years predisposed to deterioration in HRQL. Therefore we did not find support that HRQL justifies withholding ipilimumab based on age. In this dataset, pretreated patients and patients with ECOG PS ≥1 were less likely to experience impairments in global health during treatment. This may simply be due to their baseline levels being poorer. Alternatively, the patients with a higher disease burden experienced more symptomatic relief from treatment. However, this interpretation is not consistent with findings for LDH. Moreover, these observations may be incidental due to multiple testing and need to be validated in an independent patient cohort. Impaired HRQL has previously been associated with progression,^29^ and may well be associated with increasing disease-related symptoms, and/or the psychological burden of disease progression, and therapeutic failure in this study. Importantly, due to lack of a control group, this study does not address whether HRQL deteriorations were treatment related or related to disease progression or other causes. In the MDX010-20 trial, no clinically significant HRQL differences were observed between ipilimumab and the gp100 vaccine.^25^ Previous studies of HRQL in patients receiving ipilimumab have suggested distress peaking 10-12 weeks after treatment initiation,^25^^,^^26^^,^^30^ the timing of the second questionnaire in our study. As indicated by a median PFS of 2.7 months,^12^ half of patients had progressed by this point. Further, most toxicities were encountered by 10-12 weeks, supporting the timing of the HRQL assessment.

No significant association between high-grade toxicity or irAEs and deteriorations in global health was observed, in line with a phase III trial randomising adjuvant ipilimumab in stage III melanoma against placebo.^30^ Notably, the mean reported change in diarrhoea during treatment was not clinically meaningful. Only 23% of patients reported a worsening at weeks 10-12, but investigators registered diarrhoea and colitis as adverse events in 30% of patients.^12^ Possible explanations may be transient symptoms, or selective reporting by patients not believing diarrhoea was related.

We evaluated HRQL using the EORTC-QLQ-C30, the recommended assessment in advanced skin cancers.^31^ This questionnaire is nonspecific to melanoma. With diarrhoea being the obvious exception, the EORTC-QLQ-C30 does not specifically address irAEs. irAEs may affect any organ in the body and are often managed by immunosuppression.^32^ A qualitative study recognised itching, rashes, muscle stiffness, cramping, fever, and chills, as well as a protracted feeling of having a cold, to be associated with HRQL in patients receiving immune checkpoint inhibitors.^16^ In the Ipi4 trial, pruritus was recorded in 11% of patients, rash in 25%, musculoskeletal symptoms in 2%, fever in 4%, and flulike symptoms in 2%.^12^ Accordingly, HRQL issues may have been missed due to methodological shortcomings.

As compliance was low during follow-up, this HRQL report is limited to 10-12 weeks. Although most irAEs are reversible, endocrinopathies are usually persistent, potentially affecting HRQL long term. Further, a substantial proportion of patients will require immunosuppression. Yet, a recent report on HRQL in long-term survivors following ipilimumab observed no difference in global health despite lower functioning scores and higher symptoms scores compared with healthy controls.^33^

In a benefit-risk assessment, a treatment’s impact on HRQL may alter the balance. Although >50% of patients in the current trial reported maintained or less symptoms, >50% of patients experienced clinically meaningful deteriorations in global health, stressing the limitations in evaluating means in this context. Overall, however, we did not find support that ipilimumab improved HRQL during treatment in this population.

HRQL has previously been identified as prognostic in patients with metastatic melanoma.^34^ In our study, physical and role functioning, fatigue, and appetite loss were associated with OS. Combining these PROMs with previously identified risk factors was more accurately associated with OS than biological factors, or PROMs alone. To our knowledge, this is the first proposed prognostic model comprising both PROMs and biological factors in melanoma and needs validation in other populations to verify its use. For clinical decision making, it may be particularly useful to identify patients that will not achieve long-term survival, and where antitumour therapy may not be in their best interest. It would therefore be important to establish if an entity resembling group C in our dataset, with a poor median survival and no long-term survivors, can be identified. The strategy of combining PROMs with objective markers may be applicable also to other cancer forms. Importantly, PROMs offer complementary information, as compared with laboratory markers and outcomes reported by the doctor.

Despite a high compliance for the baseline questionnaire, our study has some limitations. As expected, the compliance for the second questionnaire was lower due to some patients experiencing rapid progression. Unfortunately, patients who progressed before evaluation did by protocol not reply to the second questionnaire. Therefore, this study contributes little information on the HRQL in these patients. Multiple testing of patient characteristics and HRQL indicators with respect to clinical outcomes may have provided incidental findings. Further, observations should be validated in independent patient cohorts.

Currently, ipilimumab is most frequently used in combination with nivolumab, and our findings may not be directly applicable to this clinical practice. However, ipilimumab is also administered as monotherapy after progression on PD-1 inhibitors. In our study, one in three patients had received prior therapy, although not PD-1 inhibitors. In reports on ipilimumab after progression on PD-1 inhibitors, other clinical outcomes were largely in line with ipilimumab in the first-line setting.^35^^,^^36^ Thus, our study may provide useful information on HRQL during treatment with ipilimumab in the real-world setting.

### Conclusions

In summary, increased CRP was associated with poorer HRQL. Concurring with progression for many patients, clinically meaningful and statistically significant deteriorations in HRQL were observed during treatment, supporting findings from clinical trials. Baseline HRQL was independently associated with survival and may in combination with biomarkers be valuable in prognostication, emphasising the importance of patient–doctor communication in clinical decision making.
