# Supplementary material for: Health-related quality of life in patients with advanced melanoma treated with ipilimumab: prognostic implications and changes during treatment
Source: ESMO Open. 2022 Sep 16;7(5):100588. doi: 10.1016/j.esmoop.2022.100588 (PMC9588897; doi:10.1016/j.esmoop.2022.100588)
Supplement: Supplementary Material [file mmc1.pdf]

Supplementary appendix	
Supplementary figure 1	p.2
Supplementary figure 2	p.3
Supplementary table 1	p.4
Supplementary table 2	p.5
Supplementary table 3	p.6
Supplementary table 4	p.7
Supplementary table 5	p.8
Supplementary table 6	p.9

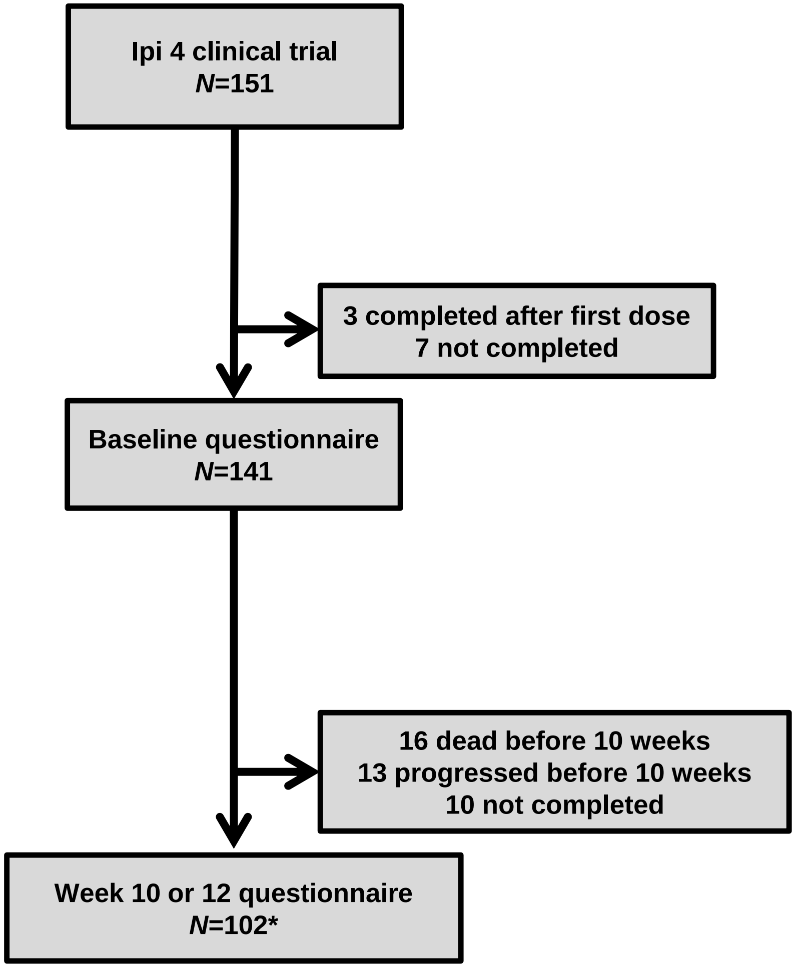

Supplementary figure 1. **Overview of patients in the Ipi4 clinical trial who completed the EORTC QLC-C30.**

The baseline questionnaire was completed by 141 patients. Three patients completed the questionnaire after the first treatment and seven patients did not complete the EORTC QLQ-C30 due to unknown reasons. At week 10 or 12, 102 patients completed the second questionnaire. Reasons for not completing the week 10 or 12 questionnaire included death (*N*=16) and progression (*N*=13) prior to week 10. Ten patients did not complete the second questionnaire due to unknown reasons. Compliance was 82% in patients alive at week 10 completing the questionnaire at week 10 or 12. This included 13 patients that had progressed prior to week 10.

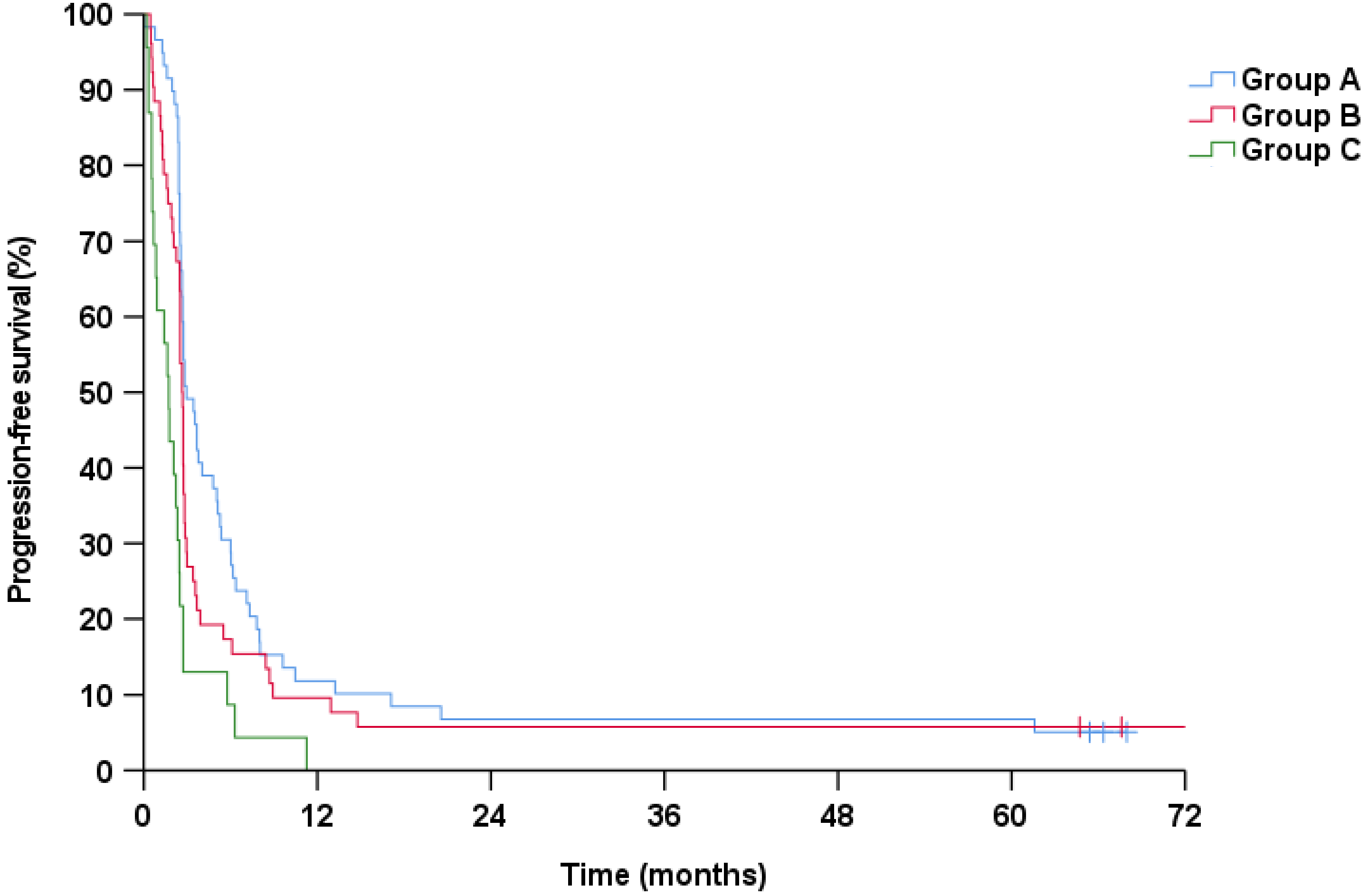

	0	12	24	36	48	60	72
A	59	7	4	4	4	4	0
B	52	5	3	3	3	3	1
C	23	0	0	0	0	0	0

Supplementary figure 2. Kaplan-Meier estimates for progression-free survival according to the prognostic index
Prognostic model combining patient-reported outcome measures with biomarkers. PFS according to number of identified risk factors; ECOG PS ≥1, LDH >ULN, CRP ≥10 mg/L, and impaired physical functioning, role functioning, fatigue and appetite loss. Patients in group A (*N*=59) harboured no risk or one factor, group B (*N*=52) two to four, and group C (*N*=23) five or more. Patients in group A had a median PFS of 3.0 months (95% CI, 2.2-3.7), patients in group B had a median PFS of 2.7 months (95% CI, 2.5-2.8), and patients in group C had a median PFS of 1.7 months (95% CI, 1.2-2.3).

Supplementary table 1. Mean baseline scores for global health and functioning scales according to baseline characteristics and biomarkers (*N*=141)

			Global health			Physical functioning			Role functioning			Emotional functioning			Cognitive functioning			Social functioning		
		N	**Mean**	**95% CI**	***p***	**Mean**	**95% CI**	***p***	**Mean**	**95% CI**	***p***	**Mean**	**95% CI**	***p***	**Mean**	**95% CI**	***p***	**Mean**	**95% CI**	***p***
**Sex**																				
	**Female**	53	70.0	63.8,76.1	0.035	84.4	79.9,88.9	0.089	78.3	71.0,85.6	0.601	79.1	73.4,84.8	0.050	93.0	90.2,95.8	0.147	76.1	69.3,82.9	0.112
	**Male**	88	77.9[a]	73.5,82.3		89.3	85.8-92.8		80.7	75.2,86.2		85.5	81.8,89.2		89.3	84.7,93.9		82.6	77.8,87.3	
**Age**																				
	**<65**	84	72.2[a]	67.4,76.9	0.075	86.1	82.3,89.9	0.239	75.6	69.6,81.6	0.020	82.5	78.3,86.8	0.672	90.5	86.8,94.2	0.265	77.0	71.5,82.5	0.051
	**>65**	57	78.8	73.3,84.3		89.5	85.5,93.5		86.0	80.1,91.9		83.9	79.1,88.7		93.3	90.7,95.9		84.8	79.6,90.0	
**ECOG PS**																				
	**0**	104	80.5	76.7,84.4	<0.001	91.9	89.4,94.5	<0.001	86.7	82.9,90.5	<0.001	85.4	82.1,88.7	0.014	94.4	92.2,96.6	<0.001	85.9	82.2,89.6	<0.001
	**≥1**	37	58.6[a]	52.5,64.6		75.0	68.6,81.3		60.4	49.6,71.1		76.6	69.1,84.0		83.8	77.3,90.3		64.0	54.8,73.1	
**M-stage**																				
	**M1a**	15	78.9	67.7,90.0	0.578	91.6	84.8,98.3	0.262	84.4	71.2,97.7	0.202	89.4	82.6,96.3	0.097	92.2	84.5,99.9	0.978	81.1	66.0,96.3	0.150
	**M1b**	22	75.0	64.3,85.7		89.4	83.0,95.8		83.3	73.7,93.0		85.2	77.1,93.4		90.9	85.5,96.4		81.1	73.1,89.1	
	**M1c**	92	75.2[a]	70.8,79.6		87.4	83.9,90.9		80.1	74.7,85.4		80.4	76.3,84.5		91.5	88.2,94.7		81.7	77.1,86.3	
	**M1d**	12	67.4	53.4,81.4		79.4	66.2,92.7		65.3	43.4,87.1		91.7	81.8,101.5		93.1	86.0,100.1		65.3	45.4,85.2	
BRAF ^{V600}**[b]**																				
	**WT**	72	76.5	71.4,81.6	0.442	88.8	85.1,92.5	0.340	84.5	78.7,90.2	0.044	80.7	76.0,85.3	0.115	90.7	86.9,94.6	0.536	80.8	74.8,86.7	0.747
	**Mutated**	65	73.7[a]	68.6,78.8		86.1	81.7,90.4		75.6	69.1,82.2		85.8	81.4,90.2		92.3	89.2,95.4		79.5	74.2,84.8	
**Prior therapy**																				
	**0**	93	75.8[a]	71.2,80.4	0.480	87.7	84.1,91.4	0.788	80.3	74.9,85.7	0.752	82.0	78.1,85.8	0.334	92.1	89.1,95.2	0.567	81.4	76.6,86.1	0.391
	**≥1**	48	73.1	67.2,79.0		86.9	82.7,91.2		78.8	71.4,86.3		85.2	79.7,90.8		90.6	86.5,94.7		77.8	70.7,84.9	
**LDH[c]**																				
	**≤ULN**	76	75.9	70.7,80.1	0.390	88.6	85.2,92.0	0.277	81.8	76.3,87.3	0.224	81.5	76.6,86.3	0.388	92.1	89.3,94.9	0.622	82.9	77.9,87.9	0.077
	**>ULN**	62	72.7[a]	67.6,77.8		85.5	80.7,90.2		76.3	69.1,83.6		84.3	80.3,88.3		90.9	86.5,95.2		75.8	69.5,82.2	
**CRP[b]**																				
	**<10 mg/L**	86	78.5[a]	73.8,83.2	0.005	90.2	86.9,93.5	0.012	84.9	80.2,89.6	0.001	84.4	80.3,88.5	0.274	92.4	89.7,95.2	0.386	85.7	81.4,89.9	<0.001
	**≥10 mg/L**	51	68.0	62.4,73.5		82.9	77.8,87.9		70.3	61.8,78.8		80.7	75.5,85.9		90.2	85.3,95.1		69.9	62.6,77.3	

[a]One patient not available [b]Four patients not available [c]Three patients not available. Abbreviations; ECOG PS=Eastern Co-operative Oncology Group performance status, M-stage=metastatic stage, LDH=lactate dehydrogenase, ULN=upper limit normal 205 U/L

Supplementary table 2. Mean baseline scores for symptoms according to baseline characteristics and biomarkers

			Fatigue			Nausea and vomiting			Pain			Insomnia			Appetite loss			Diarrhoea		
		N	**Mean**	**95% CI**	***p***	**Mean**	**95% CI**	***p***	**Mean**	**95% CI**	***p***	**Mean**	**95% CI**	***p***	**Mean**	**95% CI**	***p***	**Mean**	**95% CI**	***p***
**Sex**																				
	**Female**	53	29.6	22.5,36.7	0.135	5.0	2.6,7.5	0.673	16.1	10.9,21.3	0.298	28.3	19.4,37.2	0.003	15.7	8.6,22.8	0.519	15.1	9.0,21.2	0.063
	**Male**	88	23.5	18.9,28.1		4.4	2.4,6.3		20.8	13.2,28.3		14.8	10.4,19.2		12.9	7.6,18.2		8.7	4.9,12.5	
**Age**																				
	**<65**	84	27.2	21.7,32.8	0.363	4.8	2.6,6.9	0.812	20.4	14.7,26.2	0.146	19.0	13.3,24.8	0.660	16.3	10.4,22.2	0.186	8.7	4.8,12.6	0.083
	**>65**	57	23.6	18.4,28.8		4.4	2.3,6.5		14.0	7.6,20.4		21.1	14.0,28.1		10.5	4.7,16.4		14.6	8.8,20.4	
**ECOG PS**																				
	**0**	104	19.9	16.1,23.7	<0.001	3.0	1.5,4.6	0.001	10.3	6.7,13.8	<0.001	19.6	14.6,24.6	0.818	10.3	6.1,14.4	0.003	11.2	7.3,15.1	0.915
	**≥1**	37	42.3	33.8,50.9		9.0	5.4,12.6		39.2	28.9,49.5		20.7	11.1,30.3		24.3	13.6,35.0		10.8	4.4,17.3	
**M-stage**																				
	**M1a**	15	17.0	4.5,29.5	0.089	3.3	-0.5,7.2	0.286	16.7	3.6,29.7	0.777	20.0	3.2,36.8	0.952	8.9	-2.1,19.8	0.043	8.9	0.4,17.3	0.912
	**M1b**	22	24.7	17.2,32.3		2.3	-0.3,4.9		12.9	4.4,21.4		18.2	6.4,30.0		13.6	1.0,26.3		12.1	2.4,21.8	
	**M1c**	92	25.6	20.8,30.4		4.9	2.9,6.9		19.2	13.8,24.6		20.7	15.4,25.9		12.3	7.5,17.1		11.6	7.3,15.9	
	**M1d**	12	39.8	20.2,59.4		8.3	1.2,15.5		18.1	-3.8,39.9		16.7	-4.5,37.8		33.3	11.2,55.5		8.3	-1.2,17.9	
BRAF ^{V600a}																				
	**WT**	72	23.3	18.1,28.5	0.252	3.2	1.3,5.2	0.114	13.2	7.7,18.7	0.023	16.7	10.6,22.7	0.161	11.6	5.7,17.5	0.268	12.0	7.2,16.8	0.492
	**Mutated**	65	27.9	21.9,33.8		5.6	3.3,8.0		23.1	16.4,29.7		23.1	16.2,29.9		16.4	10.0,22.8		9.7	5.2,14.3	
**Prior therapy**																				
	**0**	93	23.8	19.1,28.4	0.159	4.1	2.2,6.1	0.380	16.8	11.6,22.1	0.520	20.1	14.8,25.3	0.894	13.6	8.3,18.9	0.831	11.1	7.0,15.2	1.000
	**≥1**	48	29.6	22.5,36.8		5.6	3.0,8.1		19.8	12.1,27.4		19.4	11.3,27.6		14.6	7.4,21.8		11.1	5.3,16.9	
**LDH**[b]																				
	**≤ULN**	76	23.7	19.1,28.3	0.140	3.5	1.9,5.1	0.090	16.9	10.9,22.9	0.538	20.6	14.3,27.0	0.691	10.5	5.2,15.8	0.057	11.4	7.0,15.8	0.974
	**>ULN**	62	29.6	22.9,36.3		6.2	3.3,9.1		19.6	13.2,26.1		18.8	12.6,25.1		18.8	11.9,25.8		11.3	6.0,16.6	
**CRP**[a]																				
	**< 10 mg/L**	86	20.9	16.4,25.5	0.001	2.5	0.9,4.1	<0.001	11.4	7.0,15.8	<0.001	19.8	13.8,25.7	0.808	7.8	3.4,12.1	<0.001	10.5	6.5,14.5	0.581
	**≥ 10 mg/L**	51	34.9	27.9,41.8		8.2	5.2,11.2		29.4	21.0,37.8		20.9	13.9,27.9		25.5	17.2,33.8		12.4	6.2,18.6	

[a]Four patients not available [b]Three patients not available. Abbreviations; ECOG PS=Eastern Co-operative Oncology Group performance status, M-stage=metastatic stage, LDH=lactate dehydrogenase, ULN=upper limit normal 205 U/L

Supplementary table 3. Change in health-related quality of life outcomes from baseline to week 10-12

	Patients answering questionnaires at baseline and week 10-12 *N*=102		Estimated by mixed linear model[a] *N*=144	
**Scales**	**Mean**	**95% CI**	**Mean**	**95% CI**
**Global health**	-13.6	(-18.1,-9.1)	-13.2	(-17.1,-9.4)
**Physical functioning**	-10.7	(-14.7,-6.7)	-10.3	(-13.4,-7.3)
**Role functioning**	-15.4	(-20.8,-9.9)	-14.2	(-18.8,-9.6)
**Emotional functioning**	-2.5	(-5.9,1.0)	-2.3	(-4.8,0.2)
**Cognitive functioning**	-7.8	(-12.7,-3.0)	-4.1	(-6.7,-1.5)
**Social functioning**	-3.8	(-7.3,-0.2)	-8.1	(-11.9,-4.2)
**Fatigue**	11.4	(6.4,16.4)	10.2	(6.2,14.1)
**Nausea and vomiting**	2.8	(0.5,5.1)	2.7	(0.3,5.2)
**Pain**	5.6	(0.6,10.5)	5.9	(1.9,9.9)
**Insomnia**	3.6	(-2.0,9.2)	3.6	(-0.8,8.0)
**Appetite loss**	12.1	(6.3,17.9)	11.9	(6.9,16.9)
**Diarrhoea**	7.5	(1.4,13.6)	7.4	(2.3,12.4)

[a]Mixed linear model incorporating all available outcomes from baseline, week 4, week 7 and week 10-12 to estimate missing data

Supplementary table 4. Mean changes in health related quality of life from baseline to 10-12 weeks by baseline characteristics and biomarkers (*N*=102)

			**Global health**			**Physical function**			**Role function**			**Fatigue**			**Appetite loss**			**Diarrhoea**		
		N		**95% CI**	***p***		**95% CI**	***p***		**95% CI**	***p***		**95% CI**	***p***		**95% CI**	***p***		**95% CI**	***p***
**Sex**																				
	**Female**	39	-11.1	-18.5,-3.8	0.398	-11.5	-18.2,-4.7	0.777	-15.0	-23.8,-6.1	0.910	12.1	3.6,20.7	0.822	12.8	2.4,23.2	0.846	10.3	-0.9,21.4	0.486
	**Male**	63	-15.1	-20.9,-9.3		-10.3	-15.4,-5.2		-15.6	-22.8,-8.4		10.9	4.6,17.3		11.6	4.5,18.7		5.8	-1.5,13.1	
**Age**																				
	**<65**	63	-11.5	-16.9,-6.1	0.252	-10.6	-15.7,-5.4	0.932	-15.1	-22.0,-8.2	0.898	15.0	8.4, 21.6	0.069	13.8	5.8,21.7	0.473	11.1	2.9,19.4	0.138
	**>65**	39	-16.9	-24.9,-8.8		-10.9	-17.6,-4.2		-15.8	-25.2,-6.4		5.6	-2.0,13.1		9.4	0.8,18.0		1.7	-7.2,10.6	
**ECOG PS**																				
	**0**	80	-16.3	-21.4,-11.1	0.023	-10.4	-15.0,-5.8	0.777	-15.6	-21.5,-9.7	0.856	13.1	7.4,18.9	0.189	11.7	5.1,18.2	0.784	6.7	0.3,13.1	0.601
	**≥1**	22	-3.8	-12.5,5.0		-11.8	-20.8,-2.9		-14.4	-29.2,0.4		5.1	-5.6,15.7		13.6	0.2,27.1		10.6	-6.7,27.9	
**M-stage**																				
	**M1a**	14	-15.5	-27.7,-3.3	0.549	-13.3	-26.0,-0.7	0.636	-14.3	-27.8,-0.8	0.480	15.1	-1.9,32.1	0.857	2.4	-6.8,11.5	0.083	21.4	2.0,40.8	0.080
	**M1b**	18	-10.2	-22.0,1.7		-14.8	-27.0,-2.6		-14.8	-27.5,-2.1		12.3	-1.0,25.7		3.7	-15.9,23.3		16.7	-3.2,36.6	
	**M1c**	63	-15.2	-21.0,-9.5		-9.6	-14.6,-4.6		-17.5	-24.9,-10.1		9.8	3.6,15.9		18.0	11.4,24.5		3.2	-3.7,10.0	
	**M1d**	7	-3.6	-27.9,20.7		-4.8	-10.6,1.1		0.0	-26.7,26.7		15.9	-9.2,40.9		0.0	-39.8,39.8		-4.8	-26.0,16.5	
***BRAF**^{V600}*[a]																				
	**WT**	52	-14.7	-21.7,-7.8	0.630	-12.3	-18.2,-6.4	0.489	-18.3	-26.9,-9.7	0.364	12.0	4.7,19.2	0.781	12.2	3.8,20.6	0.958	7.1	-2.6,16.7	0.839
	**Mutated**	48	-12.5	-18.7,-6.3		-9.4	-15.2,-3.7		-13.2	-20.2,-6.2		10.5	3.1,17.9		12.5	3.9,21.1		8.3	0.2,16.4	
**Prior therapy**																				
	**0**	65	-14.7	-19.9,-9.6	0.493	-12.5	-17.5,-7.5	0.241	-17.9	-25.3,-10.6	0.216	13.6	7.0,20.2	0.248	11.3	4.2,18.3	0.716	11.3	3.4,19.2	0.105
	**≥1**	37	-11.5	-20.2,-2.7		-7.6	-14.4,-0.7		-10.8	-19.0,-2.6		7.5	-0.3,15.3		13.5	2.9,24.1		0.9	-8.7,10.5	
**LDH**[b]																				
	**≤ULN**	62	-9.8	-14.7,-4.9	0.026	-7.8	-11.6,-4.1	0.046	-13.2	-19.4,-6.9	0.228	7.1	1.5,12.6	0.023	9.1	1.8,16.5	0.201	7.0	-0.5,14.5	0.760
	**>ULN**	37	-20.5	-29.7,-11.3		-16.4	-25.4,-7.3		-20.3	-31.4,-9.1		19.2	9.2,29.3		17.1	6.8,27.5		9.0	-2.6,20.6	
**CRP**[c]																				
	**<10 mg/L**	68	-13.1	-18.1,-8.1	0.901	-9.9	-13.9,-5.9	0.343	-16.4	-22.8,-10.0	0.750	13.6	7.9,19.3	0.223	15.7	8.4,23.0	0.088	7.8	0.1,15.6	0.880
	**≥10 mg/L**	30	-12.5	-22.3,-2.7		-14.2	-24.6,-3.8		-14.4	-26.4,-2.5		6.7	-4.5,17.9		4.4	-6.3,15.2		8.9	-2.4,20.2	

[a]2 patients not available [b]3 patients not available [c]4 patients not available Abbreviations; ECOG PS=Eastern Co-operative Oncology Group performance status, M-stage=metastatic stage, LDH=lactate dehydrogenase, ULN=upper limit normal 205 U/L, CRP=C-reactive protein

Supplementary table 5. Proportions of patients with clinical meaningful deterioration in HRQL from baseline to 10-12 weeks by baseline characteristics and biomarkers (*N*=102)

			Decreased global health			Decreased physical function			Decreased role function			Increased fatigue			Increased appetite loss			Increased diarrhea		
		N	***N***	**%**	***p***	***N***	**%**	***p***	***N***	**%**	***p***	***N***	**%**	***p***	***N***	**%**	***p***	***N***	**%**	***p***
**Sex**																				
	**Female**	39	22	**56**	0.818	21	**54**	0.649	19	**49**	0.839	17	**44**	0.810	15	**39**	0.387	10	**26**	0.557
	**Male**	63	37	**59**		31	**49**		32	**51**		29	**46**		19	**30**		13	**21**	
**Age**																				
	**<65**	63	33	**52**	0.156	33	**52**	0.719	32	**51**	0.839	31	**49**	0.289	26	**41**	0.031	17	**27**	0.173
	**>65**	39	26	**67**		19	**49**		19	**49**		15	**39**		8	**21**		6	**15**	
**ECOG PS**																				
	**0**	80	51	**64**	0.021	39	**49**	0.390	40	**50**	1.000	39	**49**	0.158	27	**34**	0.865	16	**20**	0.240
	**≥1**	22	8	**36**		13	**59**		11	**50**		7	**32**		7	**32**		7	**32**	
**M-stage**																				
	**M1a**	14	7	**50**	0.229	7	**50**	0.656	6	**43**	0.180	6	**43**	0.801	2	**14**	0.068	5	**36**	0.337
	**M1b**	18	10	**56**		9	**50**		9	**50**		8	**44**		4	**22**		6	**33**	
	**M1c**	63	39	**62**		33	**52**		34	**54**		29	**46**		26	**41**		11	**18**	
	**M1d**	7	3	**43**		3	**43**		2	**29**		3	**43**		2	**29**		1	**14**	
BRAF ^{V600}[a]																				
	**WT**	52	31	**60**	0.733	26	**50**	0.677	26	**50**	1.000	25	**48**	0.520	16	**31**	0.478	10	**19**	0.351
	**Mutated**	48	27	**56**		26	**54**		24	**50**		20	**42**		18	**38**		13	**27**	
**Prior therapy**																				
	**0**	65	44	**68**	0.008	37	**57**	0.112	37	**57**	0.064	35	**54**	0.019	20	**31**	0.467	17	**26**	0.248
	**≥1**	37	15	**41**		15	**41**		14	**38**		11	**30**		14	**38**		6	**16**	
**LDH**[b]																				
	**≤ULN**	62	36	**58**	0.892	32	**52**	0.814	32	**52**	0.980	24	**39**	0.081	20	**32**	0.769	16	**26**	0.432
	**>ULN**	37	22	**60**		20	**54**		19	**51**		21	**57**		13	**35**		7	**19**	
**CRP**[c]																				
	**<10 mg/L**	68	40	**59**	0.842	35	**52**	0.635	35	**52**	0.893	32	**47**	0.339	27	**40**	0.057	15	**22**	0.620
	**≥10 mg/L**	30	17	**57**		17	**57**		15	**50**		11	**37**		6	**20**		8	**27**	

[a]2 patients not available [b]3 patients not available [c]4 patients not available Abbreviations; ECOG PS=Eastern Co-operative Oncology Group performance status, M-stage=metastatic stage, LDH=lactate dehydrogenase, ULN=upper limit normal 205 U/L, CRP=C-reactive protein

Supplementary table 6. Cox proportional hazard modelling for progression-free survival

		Univariable (*N*=141)			***Multivariable*** (*N*=134)											
Baseline characteristics and biomarkers		**HR**	**95% CI**	***p***	**HR**	**95% CI**	***p***	**HR**	**95% CI**	***p***	**HR**	**95% CI**	***p***	**HR**	**95% CI**	***p***
**ECOG PS ≥1 vs. 0**[a]		1.23	0.83,1.81	0.307	1.02	0.67,1.56	0.922	1.05	0.69,1.59	0.831	1.11	0.72,1.72	0.636	1.11	0.74,1.67	0.612
**LDH >ULN vs. ≤ULN**[b]		1.84	1.30,2.60	<0.001	1.73	1.21,2.47	0.003	1.77	1.23,2.54	0.002	1.75	1.22,2.50	0.002	1.69	1.17,2.42	0.005
**CRP ≥10 mg/L vs. <10 mg/L**[c]		1.69	1.18,2.41	0.004	1.48	1.02,2.15	0.039	1.34	0.91,1.98	0.145	1.47	1.01,2.14	0.042	1.35	0.92,1.98	0.125
Functioning scales[d]	≤66.7/>66.7[e]															
**Global health/QoL**[a]	38/61	1.28	0.90,1.82	0.173												
**Physical functioning**	15/85	1.85	1.15,2.96	0.011	1.86	1.12,3.08	0.016									
**Role functioning**	35/65	1.84	1.29,2.62	<0.001				1.78	1.22,2.60	0.003						
**Emotional functioning**	20/80	0.89	0.57,1.39	0.612												
**Social functioning**	36/64	1.11	0.77,1.58	0.587												
**Cognitive functioning**	11/89	0.89	0.51,1.55	0.681												
Symptoms scales[f]	≥33.3/<33.3[g]															
**Fatigue**	45/55	1.20	0.85,1.68	0.305							1.09	0.74,1.58	0.672			
**Nausea and vomiting**	4/96	1.77	0.72,4.35	0.213												
**Pain**	31/69	1.10	0.76,1.59	0.617												
**Insomnia**	44/56	1.10	0.78,1.55	0.582												
**Appetite loss**	28/72	1.83	1.26,2.67	0.002										1.58	1.06,2.35	0.025
**Diarrhoea**	27/73	1.02	0.69,1.50	0.914												

[a]One patient not available [b]Three patients not available [c]Four patients not available [d]Score ≤66.7 vs. >66.7 [e]Percentages of patients with HRQL ≤66.7 />66.7 [f]Score ≥33.3 vs. <33.3 [g]Percentages of patients with HRQL ≥33.3/<33.3 Patients with missing data were excluded from analysis Abbreviations; HR; hazard ratio, CI; confidence interval; ECOG PS; Eastern Cooperation Oncology Group performance status, ULN; upper limit normal (205 U/L).
